# Supplementary material for: RIG‐I antiviral signaling drives interleukin‐23 production and psoriasis‐like skin disease
Source: EMBO Mol Med. 2017 Apr 4;9(5):589–604. doi: 10.15252/emmm.201607027 (PMC5412807; doi:10.15252/emmm.201607027)
Supplement: Supplementary file 1 — Expanded View Figures PDF [file EMMM-9-589-s001.pdf]

Expanded View Figures

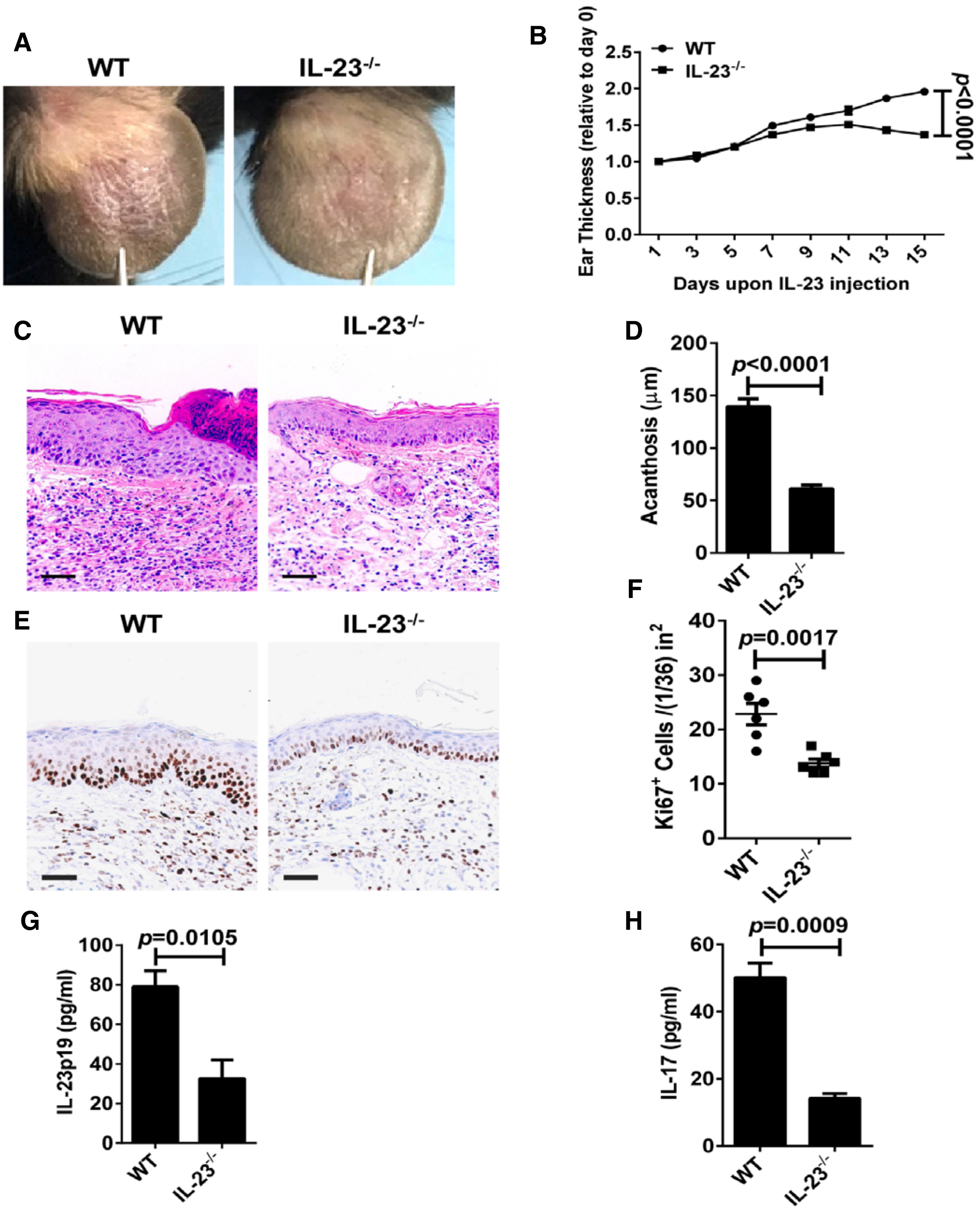

Figure EV1. IL-23-induced psoriasis-like skin disease is ameliorated in IL-23$^{-/-}$ mice.

A Representative photographs of the ears of WT mice (left panel) and IL-23$^{-/-}$ mice (right panel) after intradermal injection with IL-23 (500 ng) on every other day for 8 times, n = 6 per group.

B The ear thickness of WT and IL-23$^{-/-}$ mice on the indicated day presented relative to day 0. Significant differences are indicated: one-way ANOVA, n = 6 per group (mean ± SEM).

C Representative H&E staining of the ears treated as in (A), n = 6 per group. Scale bar: 50 μm.

D Acanthosis of WT and IL-23$^{-/-}$ mice treated with IL-23. Significant differences are indicated: two-tailed Student's t-test, n = 6 per group (mean ± SEM).

E Representative immunostaining of Ki67 in ear skin derived from WT and IL-23$^{-/-}$ mice treated with IL-23 n = 6 per group. Scale bar: 50 μm.

F Quantitation of Ki67^{+} cells in ear skin derived from WT and IL-23$^{-/-}$ mice treated with IL-23. Significant differences are indicated: two-tailed Student's t-test, n = 6 per group (mean ± SEM).

G, H ELISA detection of IL-23p19 (G) and IL-17 (H) protein levels in supernatants of ear skin homogenates derived from indicated groups. Significant differences are indicated: two-tailed Student's t-test, n = 3–5 per group (mean ± SEM).

© 2017 The Authors

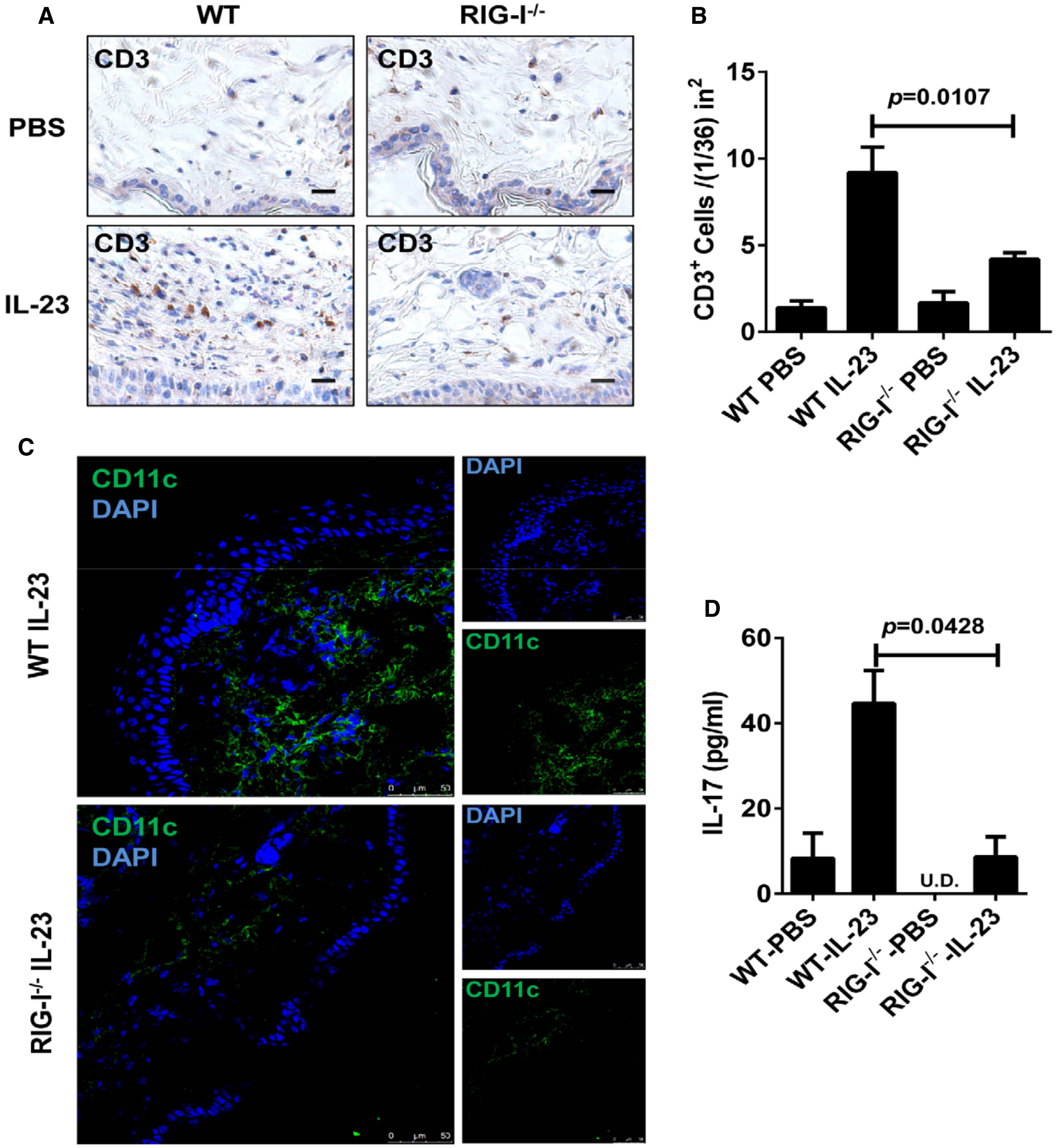

Figure EV2. Inflammatory cell infiltrates and IL-17 expression levels in IL-23-induced mouse model.

A Representative immunostaining of CD3 in ear skin derived from WT and RIG-I$^{-/-}$ mice treated with PBS or IL-23, n = 3–5 per group. Scale bar: 20 μm.

B Quantitation of CD3^{+} cells in ear skin derived from WT and RIG-I$^{-/-}$ mice treated with PBS or IL-23. Significant differences are indicated: two-tailed Student's t-test, n = 3–5 per group (mean ± SEM).

C Representative immunofluorescence staining of CD11c in ear skin derived from WT and RIG-I$^{-/-}$ mice treated with IL-23, n = 3–5 per group. Scale bar: 50 μm.

D ELISA detection of IL-17 protein levels in supernatants of ear skin homogenates derived from indicated groups. Significant differences are indicated: two-tailed Student's t-test, n = 3 per group (mean ± SEM).

 © 2017 The Authors

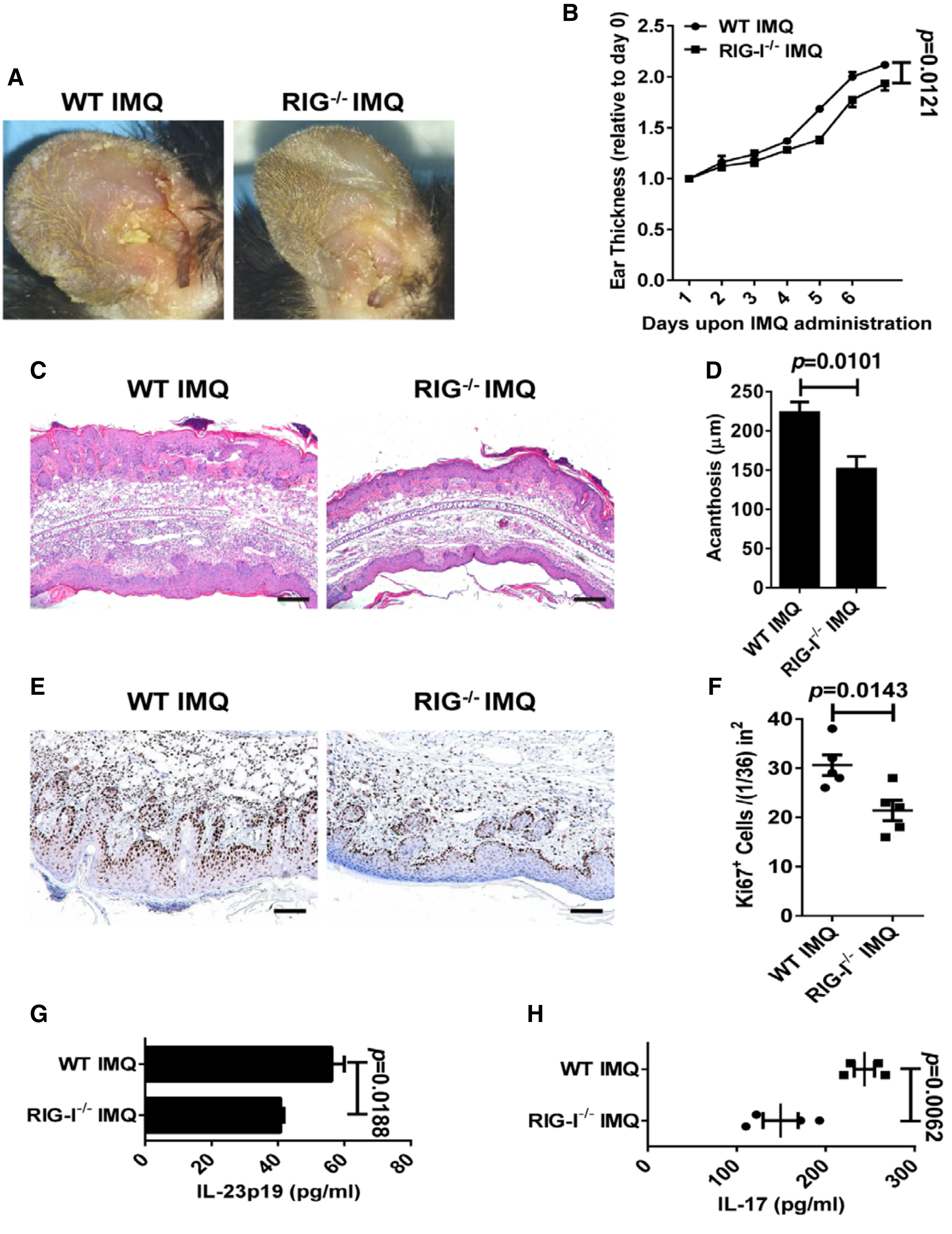

Figure EV3. IMQ-induced psoriasis-like skin disease is attenuated in RIG-I$^{-/-}$ mice.

A Representative photographs of the ears of WT mice (left panel) and RIG-I$^{-/-}$ mice (right panel) after administration of imiquimod (IMQ) for 7 days, n = 4 per group.

B The ear thickness of WT and RIG-I$^{-/-}$ mice on the indicated day presented relative to day 0. Significant differences are indicated: one-way ANOVA, n = 4 per group (mean ± SEM).

C Representative H&E staining of the ears treated as in (A), n = 4 per group. Scale bar: 200 μm.

D Acanthosis of WT and RIG-I$^{-/-}$ mice treated with imiquimod. Significant differences are indicated: two-tailed Student's t-test, n = 5 per group (mean ± SEM).

E Representative immunostaining of Ki67 in ear skin derived from WT and RIG-I$^{-/-}$ mice treated with imiquimod, n = 5 per group. Scale bar: 100 μm.

F Quantitation of Ki67^{+} cells in ear skin derived from WT and RIG-I$^{-/-}$ mice treated with imiquimod. Significant differences are indicated: two-tailed Student's t-test, n = 5 per group (mean ± SEM).

G, H ELISA detection of IL-23p19 (G) and IL-17 (H) protein levels in supernatants of ear skin homogenates derived from indicated groups. Significant differences are indicated: two-tailed Student's t-test, n = 3–4 per group (mean ± SEM).

© 2017 The Authors

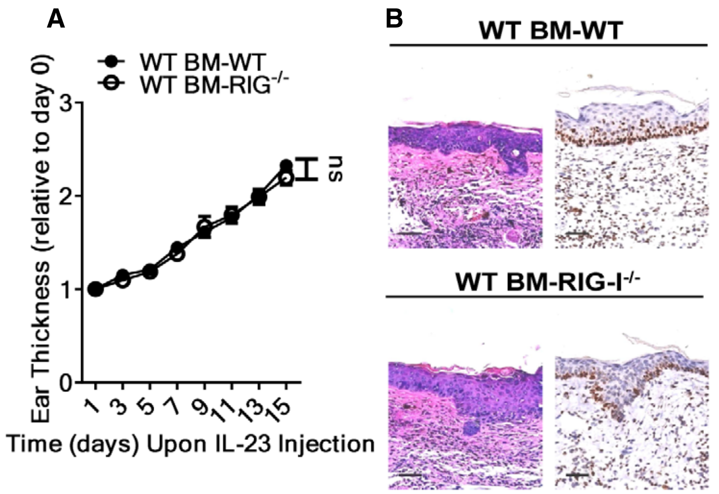

Figure EV4. RIG-I expression in non-haematopoietic cells is not required for IL-23-induced psoriasis-like skin inflammation.

A Lethally irradiated WT and RIG-I$^{-/-}$ mice were adoptively transferred with WT bone marrow (BM) cells, and the generated chimeric mice were subjected to IL-23-induced psoriasis-like skin inflammation. Data are presented on the indicated day relative to day 0. Significant differences are indicated: one-way ANOVA, n = 5 per group (mean ± SEM).

B Representative H&E staining and Ki67 immunostaining of the ears treated as in (A), n = 5 per group. Scale bar: 50 μm.

C, D Acanthosis (C) and dermal cellular infiltrates (D) of WT BM-WT or WT BM-RIG-I$^{-/-}$ mice treated with IL-23. Significant differences are indicated: two-tailed Student's t-test, n = 5 per group (mean ± SEM).

 © 2017 The Authors

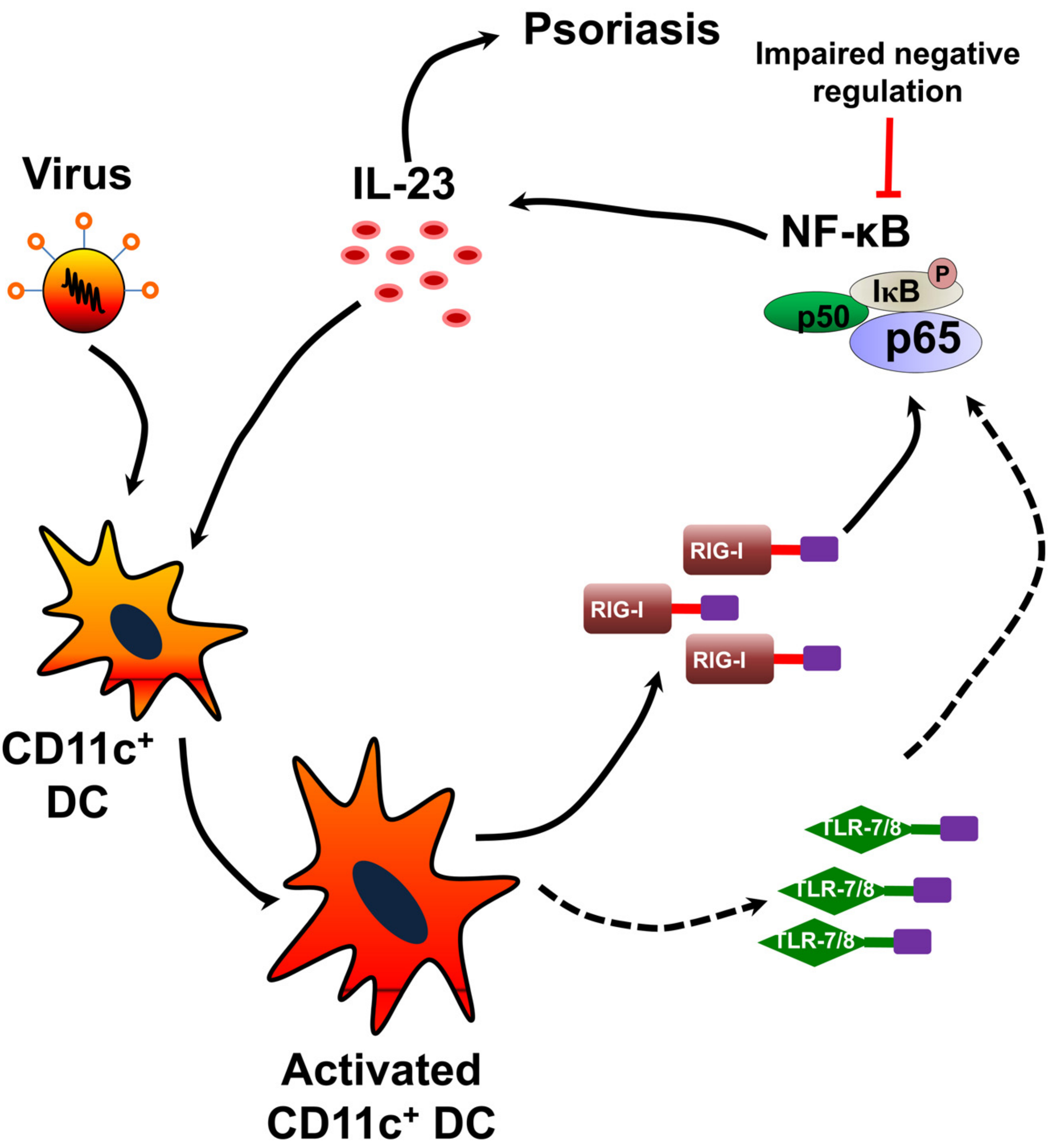

Figure EV5. Schematic diagram of how the antiviral signaling mediates psoriasis pathogenesis.
In genetically predisposed individuals, the virus infection causes the activation of TLR-7/8 and/or RIG-I, and subsequently triggers IL-23 release by CD11c$^+$ DCs via the NF-κB pathway. Genetic mutations in NF-κB-related genes result in an impaired negative regulation of its proinflammatory activity accompanied by uncontrolled IL-23 release, thus leading to psoriasis.

© 2017 The Authors
