# Supplementary material for: RIG‐I antiviral signaling drives interleukin‐23 production and psoriasis‐like skin disease
Source: EMBO Mol Med. 2017 Apr 4;9(5):589–604. doi: 10.15252/emmm.201607027 (PMC5412807; doi:10.15252/emmm.201607027)
Supplement: Supplementary file 2 — Table EV1 [file EMMM-9-589-s002.docx]

**Table EV1**

**Information for patients with psoriasis vulgaris**

| **Age/**  **Gender** | **H score** | **Sample ID** | **Age/**  **Gender** | **H score** | **Sample ID** | **Age/**  **Gender** | **H score** | **Sample ID** | **Age/**  **Gender** | **H score** |
| --- | --- | --- | --- | --- | --- | --- | --- | --- | --- | --- |
| 22/F | 153.33 | **22** | 24/M | 165.40 | **43** | 23/M | 150 | **64** | 22/M | 150 |
| 11/M | 132.67 | **23** | 16/F | 111.57 | **44** | 19/M | 137.22 | **65** | 18/M | 167.62 |
| 51/M | 172.02 | **24** | 14/F | 176.39 | **45** | 25/M | 129.17 | **66** | 31/F | 152.22 |
| 13/M | 160.48 | **25** | 28/F | 168.10 | **46** | 24/M | 141.07 | **67** | 29/M | 134.76 |
| 12/F | 208.33 | **26** | 31/F | 198.02 | **47** | 38/F | 148.89 | **68** | 38/M | 135.98 |
| 29/M | 138.73 | **27** | 12/M | 155.82 | **48** | 54/F | 166.11 | **69** | 21/F | 129.43 |
| 59/M | 156.14 | **28** | 14/F | 148.69 | **49** | 40/M | 150 | **70** | 42/M | 142.06 |
| 22/M | 159.26 | **29** | 25/M | 154.56 | **50** | 56/F | 167.68 | **71** | 46/M | 132.381 |
| 37/M | 138.69 | **30** | 29/M | 157.67 | **51** | 30/M | 199.54 | **72** | 25/F | 148.61 |
| 31/F | 162.74 | **31** | 42/F | 153.33 | **52** | 29/F | 139.90 | **73** | 26/M | 150.22 |
| 5/F | 137.30 | **32** | 60/F | 135.45 | **53** | 45/M | 122.49 | **74** | 20/F | 160 |
| 60/M | 132.59 | **33** | 40/M | 126.98 | **54** | 22/F | 108.33 | **75** | 42/M | 147.20 |
| 55/M | 146.03 | **34** | 10/F | 189.44 | **55** | 31/F | 118.65 | **76** | 25/F | 165.95 |
| 55/M | 153.77 | **35** | 57/M | 157.41 | **56** | 45/M | 153.33 | **77** | 29/F | 152.22 |
| 31/M | 245.63 | **36** | 24/M | 153.33 | **57** | 30/M | 121.75 | **78** | 59/F | 153.17 |
| 75/M | 173.81 | **37** | 34/M | 140 | **58** | 42/M | 144.44 | **79** | 25/F | 146.79 |
| 55/M | 172.22 | **38** | 8/F | 129.63 | **59** | 48/M | 161.67 | **80** | 42/M | 174.29 |
| 22/M | 130.69 | **39** | 42/M | 150.07 | **60** | 27/M | 104.76 | **81** | 31/F | 106.67 |
| 52/M | 137.5 | **40** | 28/M | 154.29 | **61** | 30/F | 156.94 | **82** | 51/M | 188.69 |
| 17/M | 139.52 | **41** | 21/M | 164.81 | **62** | 63/M | 145.19 | **83** | 25/F | 153.17 |
| 35/F | 128.56 | **42** | 18/F | 155.71 | **63** | 20/M | 151.79 | **84** | 18/F | 125 |

All patients were clinically diagnosed as psoriasis vulgaris. M, male; F, female.
