# Supplementary material for: RIG‐I antiviral signaling drives interleukin‐23 production and psoriasis‐like skin disease
Source: EMBO Mol Med. 2017 Apr 4;9(5):589–604. doi: 10.15252/emmm.201607027 (PMC5412807; doi:10.15252/emmm.201607027)
Supplement: Supplementary file 3 — Table EV2 [file EMMM-9-589-s003.docx]

**Table EV2**

**Primer sequences and siRNA sequences are listed below.**

| **Molecule** | **Primer** | **Sequence** |
| --- | --- | --- |
| Mouse RIG-I | forward | 5’-AAGAGCCAGAGTGTCAGAATCT-3’ |
|  | reverse | 5’-AGCTCCAGTTGGTAATTTCTTGG-3’ |
| Human RIG-I | forward | 5’-CTGGACCCTACCTACATCCTG-3’ |
|  | reverse | 5’-GGCATCCAAAAAGCCACGG-3’ |
| Mouse IL-23p19 | forward | 5’-GGTGGCTCAGGGAAATGT-3’ |
|  | reverse | 5’-GACAGAGCAGGCAGGTACAG-3’ |
| Mouse IL-23p19 promoter  (Luciferase Reporter Assay) | forward | 5’-CCGCTCGAGAGGCATGAGTTCCAGGACA-3’ |
|  | reverse | 5'-CCCAAGCTTTGTTCCCTGCTTCTCAGATC-3' |
|  | site-specific mutant | 5'-ATGATGTAGGGAGTTTCATAAAACCTGCTCTGAG-3' |
| Mouse IL-23p19 promoter  (ChIP) | forward | 5'-GCCTCTAGCCACAACAACCT-3' |
|  | reverse | 5'-TGACTCAGAGCAGGTGGGAT-3' |
| siRNA-1822 (p65) | forward | 5'-GGAGUACCCUGAAGCUAUATT-3' |
|  | reverse | 3'-TTCCUCAUGGGACUUCGAUAU-5' |
| siRNA-1254 (p65) | forward | 5'-GGACCUAUGAGACCUUCAATT-3' |
|  | reverse | 3'-TTCCUGGAUACUCUGGAAGUU-5' |
| siRNA-396 (p65) | forward | 5'-GGCCUUAUGUGGAGAUCAUTT-3' |
|  | reverse | 3'-TTCCGGAAUACACCUCUAGUA-5' |
| siRNA-744 ( IRF-3) | forward | 5'-GGCUAUUGUUUCUGAUCCUTT -3' |
|  | reverse | 3'-TTCCGAUAACAAAGACUAGGA-5' |
| siRNA-526 ( IRF-3) | forward | 5'-GGAAAGAAGUGUUGCGGUUTT-3' |
|  | reverse | 3'-TTCCUUUCUUCACACGCCAA-5' |
| siRNA-1346 ( IRF-3) | forward | 5'-GGUUGUUCCUACAUGUCUUTT-3' |
|  | reverse | 3'-TTCCAACAAGGAUGUACAGAA-5' |
| siRNA-1526 ( IRF-7) | forward | 5'-GCACUUUCUUCCGAGAACUTT-3' |
|  | reverse | 3'-TTCGUGAAAGAAGGCUCUUGA-5' |
| siRNA-1168 ( IRF-7) | forward | 5'-CUGGAUGUGACCAUCAUGUTT-3' |
|  | reverse | 3'-TTGACCUACACUGGUAGUACA-5' |
| siRNA-759 ( IRF-7) | forward | 5'-CUUGCGCCAAGACAAUUCATT-3' |
|  | reverse | 3'-TTGAACGCGGUUCUGUUAAGU-5' |
