# Supplementary figures and images for: RIG‐I antiviral signaling drives interleukin‐23 production and psoriasis‐like skin disease
Source: EMBO Mol Med. 2017 Apr 4;9(5):589–604. doi: 10.15252/emmm.201607027 (PMC5412807; doi:10.15252/emmm.201607027)

**Figure 1G**

**RIG-I**

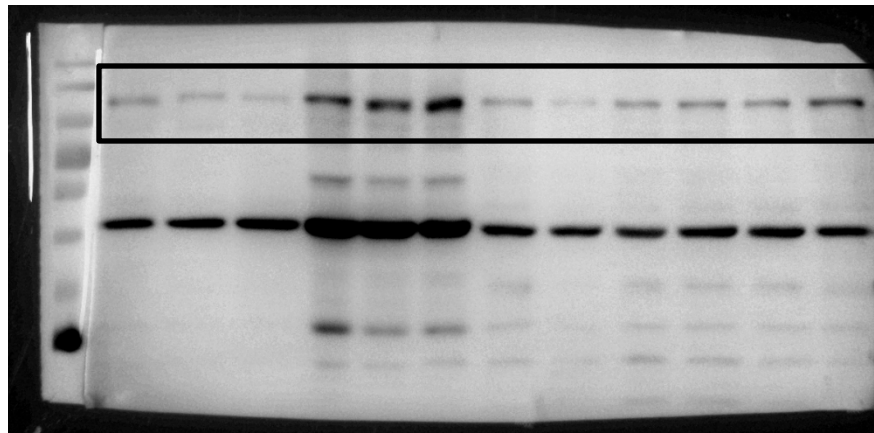

**p-I $\kappa$ B- $\alpha$**

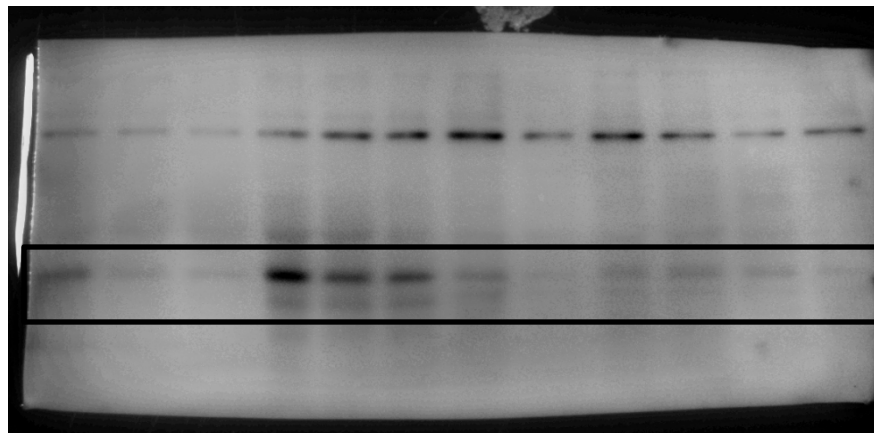

**$\beta$ -actin**

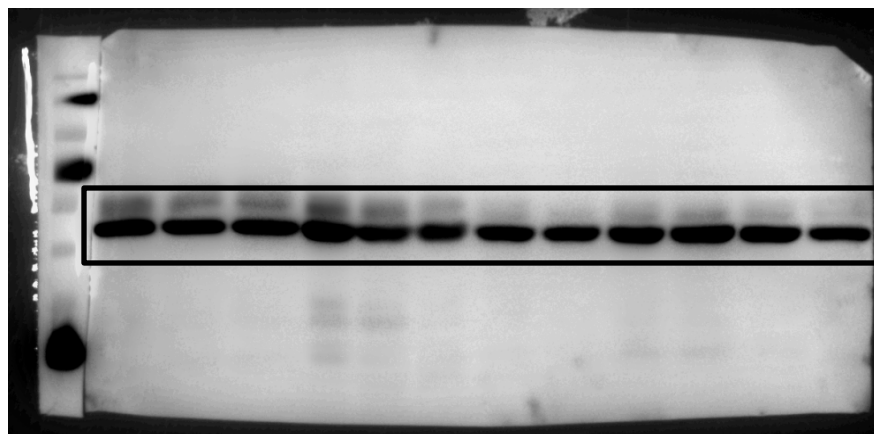

Supplement: Supplementary file 5 — Source Data for Figure 1 [file EMMM-9-589-s004.pdf]

**Figure 3E**

**RIG-I**

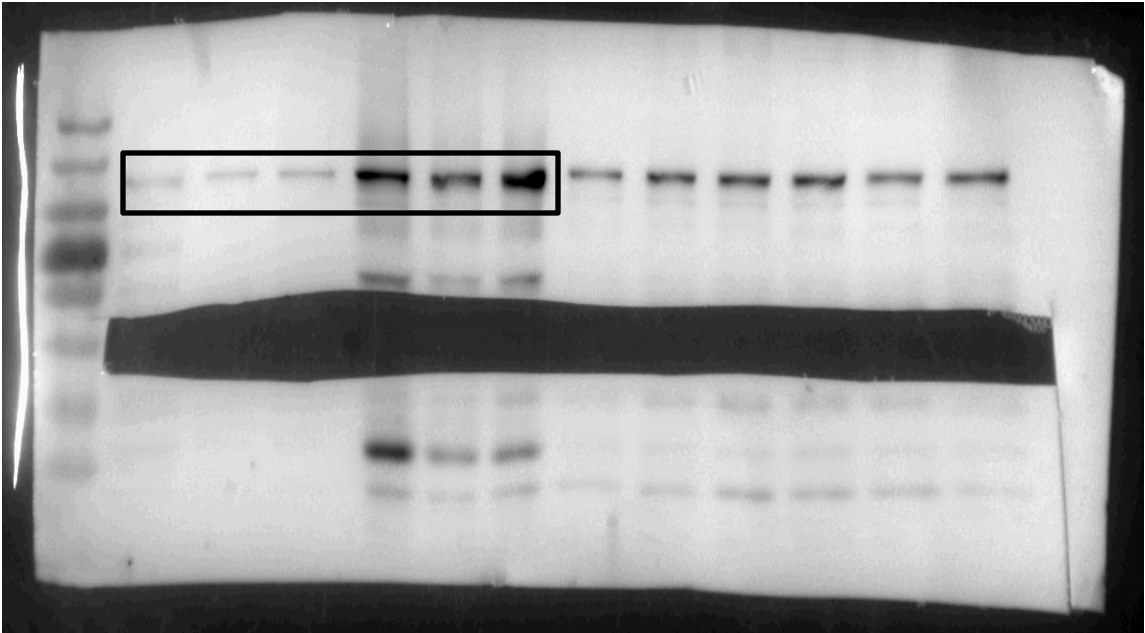

**β-actin**

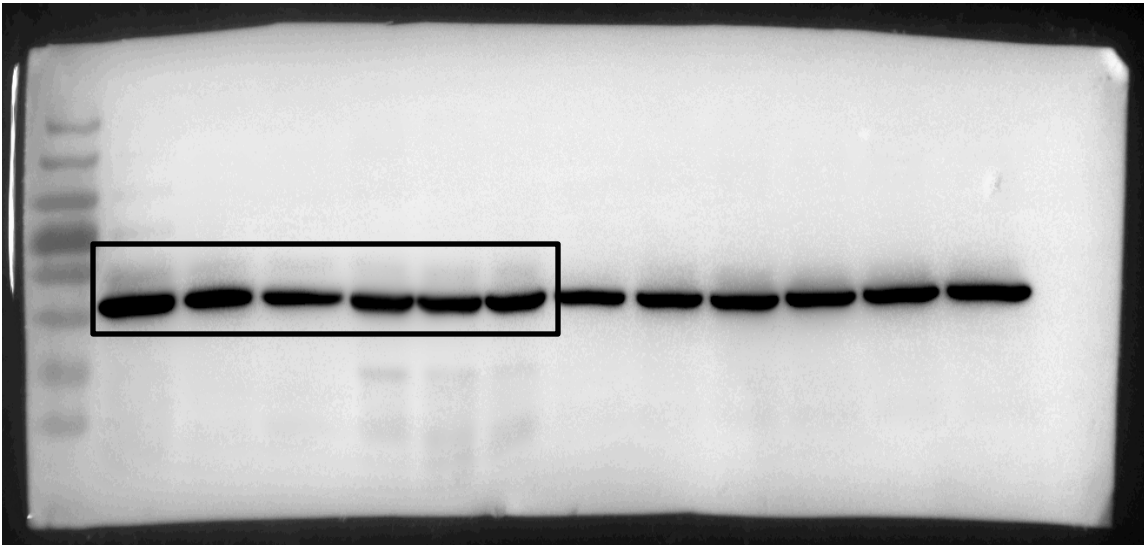

**Figure 3F**

**RIG-I**

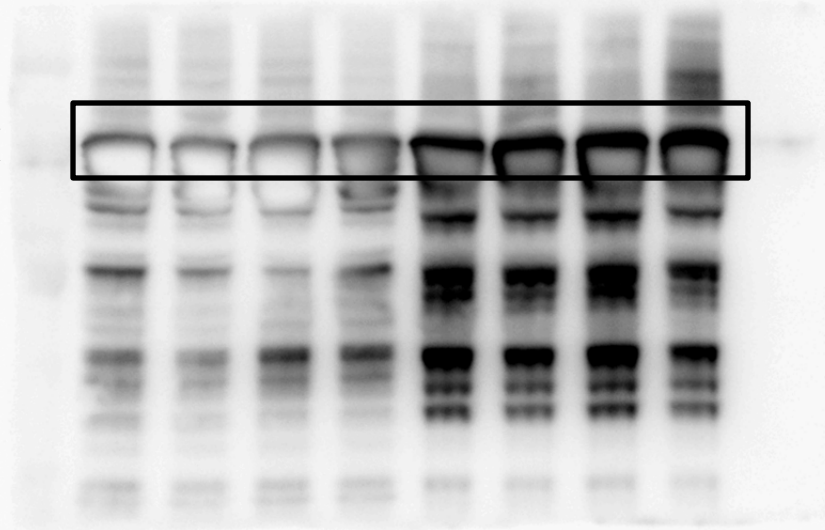

**$\beta$ -actin**

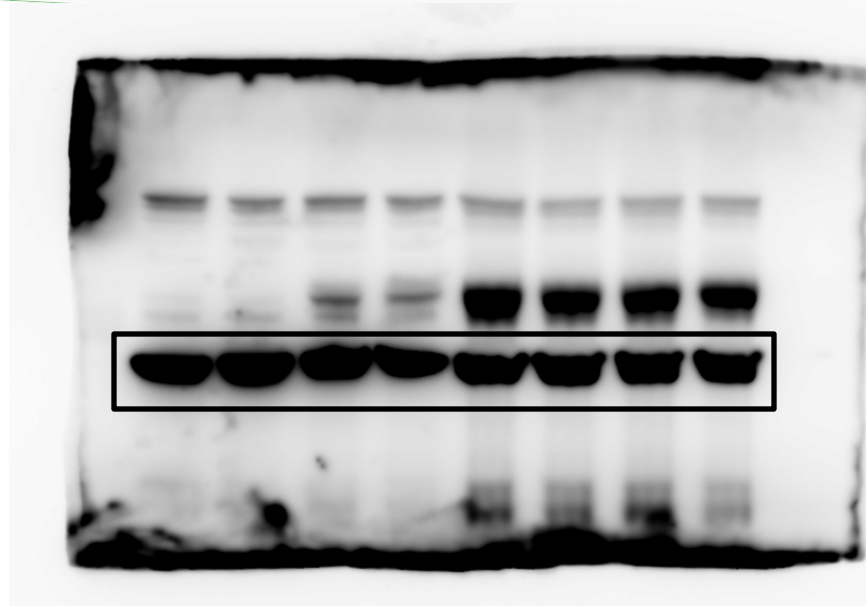

Supplement: Supplementary file 6 — Source Data for Figure 3 [file EMMM-9-589-s005.pdf]

**Figure 4G**

**RIG-I**

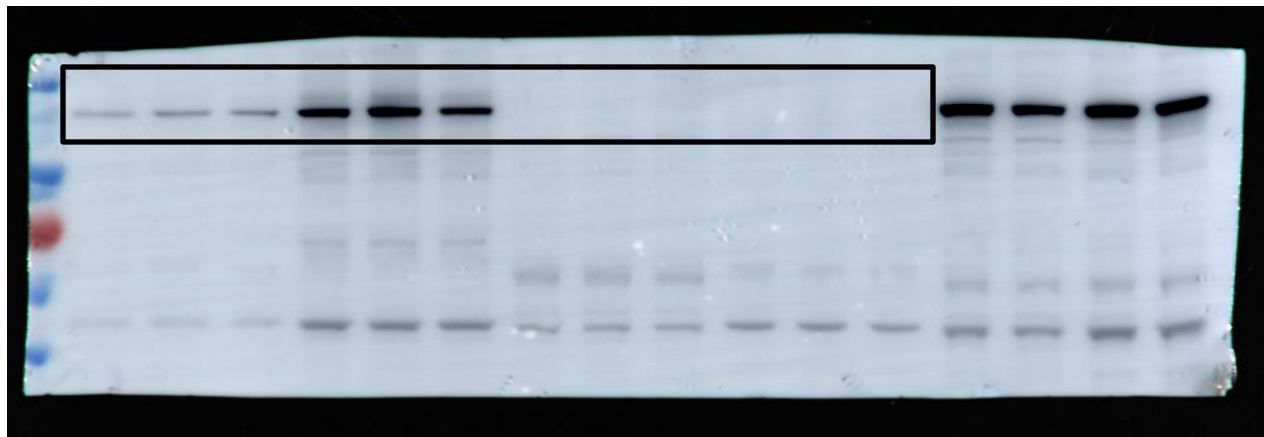

**p-I $\kappa$ B- $\alpha$**

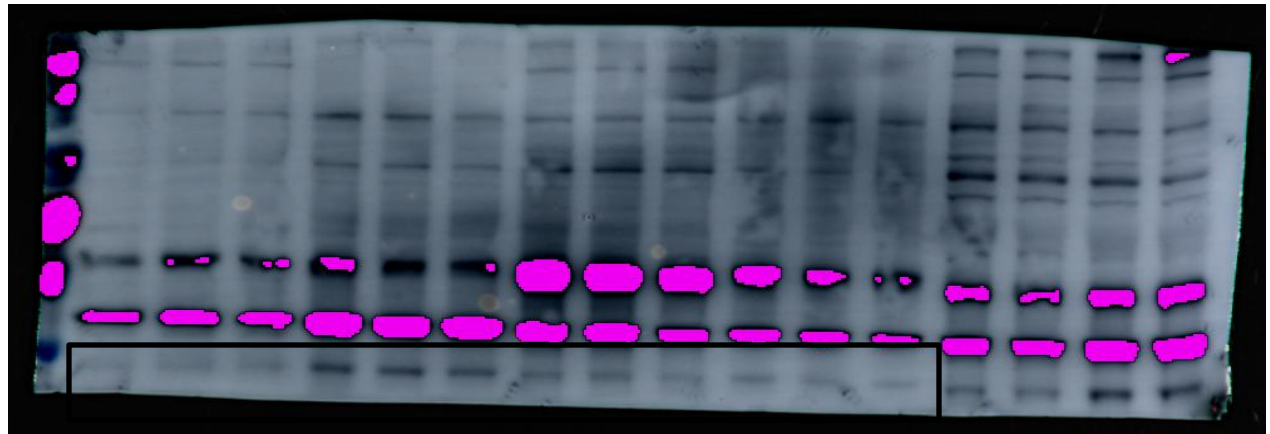

**$\beta$ -actin**

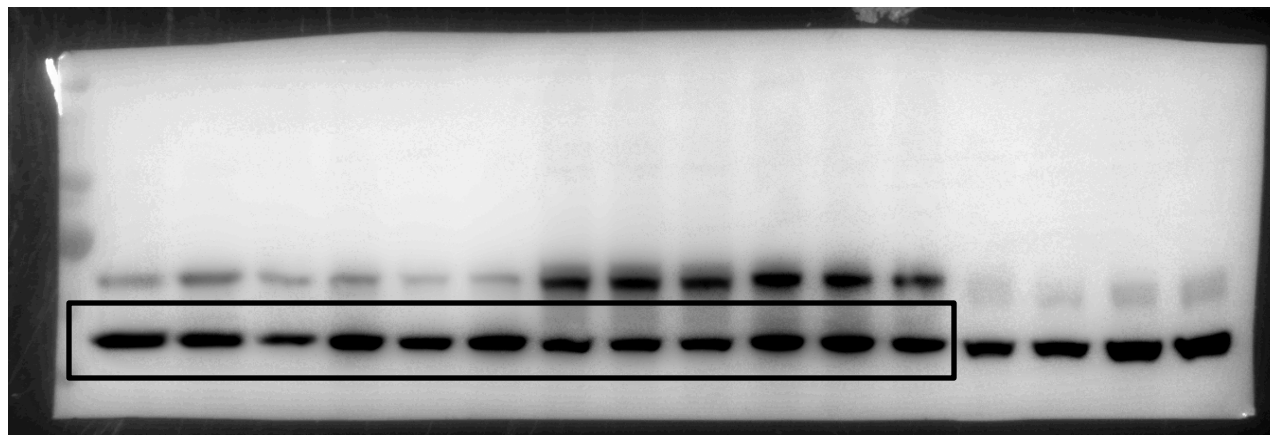

Supplement: Supplementary file 7 — Source Data for Figure 4 [file EMMM-9-589-s006.pdf]

Figure 7A

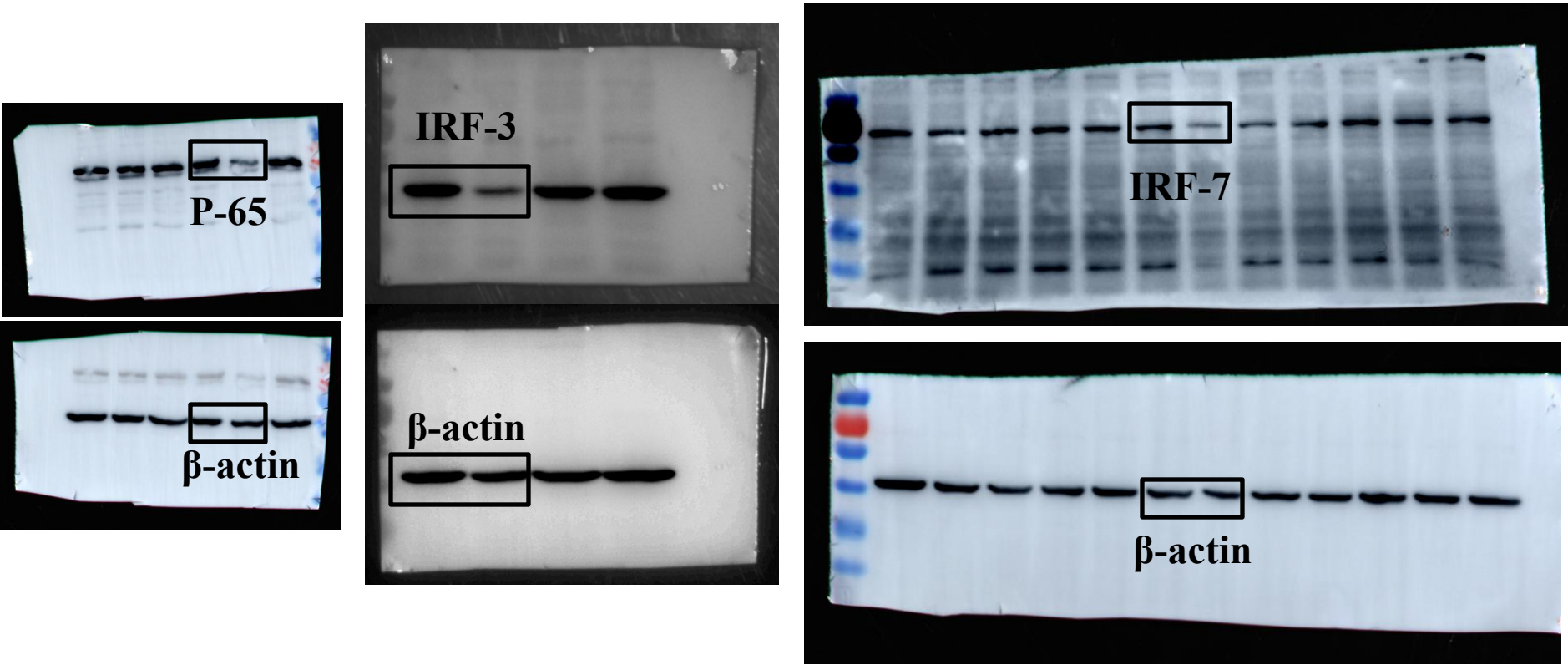

Supplement: Supplementary file 8 — Source Data for Figure 7 [file EMMM-9-589-s007.pdf]
